# Supplementary material for: Resolving the full spectrum of human genome variation using Linked-Reads
Source: Genome Res. 2019 Apr;29(4):635–45. doi: 10.1101/gr.234443.118 (PMC6442396; doi:10.1101/gr.234443.118)
Supplement: Supplemental Material [file supp_gr.234443.118_Supplemental_longranger-2.2.zip › longranger-2.2.tar.bz2!/lr2.2-public/lib/stan/cmdstan/stan/doc/api/html/check__nonzero__size_8hpp_source.html]

Stan: src/stan/math/error\_handling/matrix/check\_nonzero\_size.hpp Source File


|  |  |
| --- | --- |
|  | Stan  2.5.0  probability, sampling & optimization |


- Main Page
- Related Pages
- Namespaces
- Classes
- Files

- File List
- File Members

All Classes Namespaces Files Functions Variables Typedefs Enumerations Enumerator Friends Macros Pages

- src
- stan
- math
- error\_handling
- matrix

check\_nonzero\_size.hpp

Go to the documentation of this file.

1 #ifndef STAN\_\_MATH\_\_ERROR\_HANDLING\_\_MATRIX\_\_CHECK\_NONZERO\_SIZE\_HPP

2 #define STAN\_\_MATH\_\_ERROR\_HANDLING\_\_MATRIX\_\_CHECK\_NONZERO\_SIZE\_HPP

3

4 #include <string>

5 #include <typeinfo>

6

7 #include <stan/math/error\_handling/dom\_err.hpp>

8 #include <stan/math/matrix/meta/index\_type.hpp>

9 #include <stan/meta/traits.hpp>

10

11 namespace stan {

12

13  namespace math {

14

32  template <typename T\_y, typename T\_result>

33  inline bool check\_nonzero\_size(const char\* function,

34  const T\_y& y,

35  const char\* name,

36  T\_result\* result) {

37  typedef typename index\_type<T\_y>::type size\_t;

38  if (y.size() > 0)

39  return true;

40

41  return dom\_err(function, size\_t(),

42  name, " has size %1%, but must have a non-zero size","",

43  result);

44  }

45

46  }

47 }

48 #endif

dom\_err.hpp

stan::math::dom\_err

bool dom\_err(const char \*function, const T &y, const char \*name, const char \*error\_msg, const T\_msg error\_msg2, T\_result \*result)

**Definition:** dom\_err.hpp:36

traits.hpp

stan::math::index\_type

Primary template class for the metaprogram to compute the index type of a container.

**Definition:** index\_type.hpp:21

index\_type.hpp

stan::math::check\_nonzero\_size

bool check\_nonzero\_size(const char \*function, const T\_y &y, const char \*name, T\_result \*result)

Return true if the specified matrix/vector is of non-zero size.

**Definition:** check\_nonzero\_size.hpp:33


---

[ Stan Home Page ]

*© 2011–2014,
Stan Development Team.*
